# 肠道菌群失调与肺癌：机制解析与临床应用

**DOI:** 10.3779/j.issn.1009-3419.2025.106.02

**Published:** 2025-01-20

**Authors:** Liangyuan CHEN, Yiqing XIE, Chong LI

**Affiliations:** 213000 常州，南京医科大学/常州市第一人民医院呼吸内科; Department of Respiratory Medicine, Changzhou First People's Hospital/Nanjing Medical University, Changzhou 213000, China

**Keywords:** 肺肿瘤, 肠道菌群, 肺肠轴, 机制, 炎症, 免疫, 代谢, 化疗, Lung neoplasms, Intestinal flora, Lung-gut axis, Mechanism, Inflammation, Immunity, Metabolism, Chemotherapy

## Abstract

肺癌作为全球致死性最高的恶性肿瘤，每年新增病例数以百万计。尽管手术、靶向治疗和免疫治疗技术快速发展，极大改善了患者的生存状况，然而其总体5年生存率仍较低。近年来，研究发现肠道菌群在维持宿主健康中起到至关重要的作用，并通过多种机制与肺癌的发生发展密切相关。本文从肠道菌群的免疫调控、代谢调节及其在肺癌治疗中的应用等方面，对肠道菌群在肺癌中的作用机制进行了系统分析，展望了其在临床应用中的潜力。

## Abstract (en)

Lung cancer is the deadliest form of cancer globally, with millions of new cases diagnosed each year. Although rapid advancements in surgical techniques, targeted therapies, and immunotherapy have significantly improved patient outcomes, the overall 5-year survival rate remains disappointingly low. Recent studies have highlighted the vital role of gut microbiota in maintaining host health and its close association with the onset and progression of lung cancer through various mechanisms. This article provides a systematic analysis of the role of gut microbiota in lung cancer, focusing on its immunomodulatory and metabolic functions, as well as its potential applications in treatment, while also exploring its prospects for clinical use.

肺癌是全球范围内死亡率最高的恶性肿瘤，2022年全球新发肺癌患者数量大约为248.1万，而肺癌患者死亡人数则高达180万，严重威胁公共卫生安全。尽管手术、靶向治疗和免疫治疗等技术取得了显著进展，肺癌的5年生存率仍仅为28.7%，亟需更深入的机制研究来探索新的诊疗策略。近年来，肠道菌群对多种疾病的影响得以证实，研究^[1,2]^表明肠道菌群在肺癌的发生、发展及治疗中可能发挥重要作用。本文旨在系统总结肠道菌群与肺癌的相关机制，并探讨其在临床应用中的潜在价值。

## 1 人体菌群与肺癌

人体菌群分布于多个器官，包括肺、口腔及肠道，菌群失调已被证明与癌症的发生密切相关。研究^[3,4]^表明，肺癌患者的肺部菌群发生了显著的变化，尤其是链球菌和葡萄球菌的丰度增加，这可能是通过DNA损伤及增加基因组不稳定性促进肿瘤的发生。在口腔菌群中，拟杆菌门的丰度与肺癌的风险呈负相关，而厚壁菌门丰度与肺癌风险呈正相关^[5]^。此外，肠道菌群的失调则进一步影响免疫反应和炎症状态，从而促进肺癌发展。通过16S rRNA测序分析发现，肺癌患者的肠道微生物群呈现出多样性下降的现象。肠球菌的丰度增加，而有益菌如双歧杆菌则显著减少，这些变化不仅可能促进肺癌的发生，还可能作为早期生物标志物，用于肺癌的早期诊断和筛查^[6,7]^。

## 2 肠道菌群影响肺癌发生发展的机制

肠道菌群影响肺癌进展的机制见图1。

**图1:**
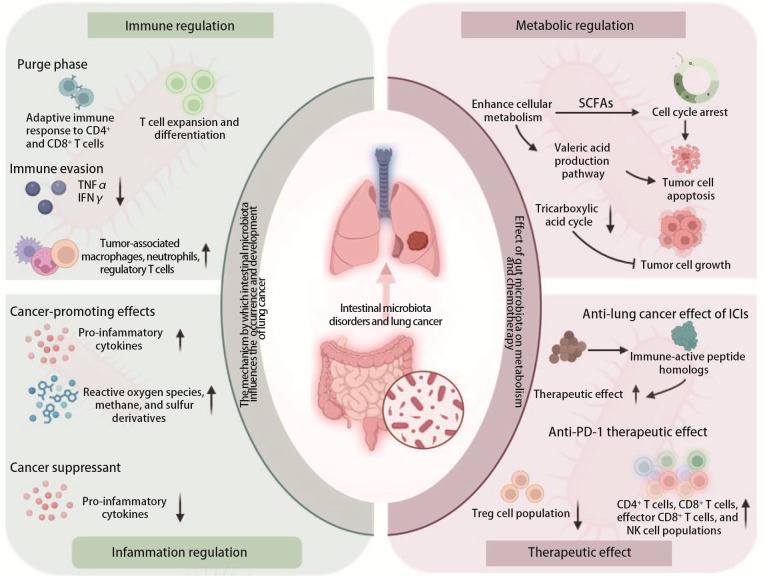
肠道菌群影响肺癌进展的机制概要图

### 2.1 免疫调控作用

肠道菌群通过调节宿主的免疫系统，在肺癌免疫监视和免疫逃逸机制中发挥双重作用。肺癌的免疫编辑被视为一个动态演变的连续过程，涵盖三个核心阶段：首先是清除阶段，随后是免疫与肿瘤的平衡期，最终是肿瘤逃逸免疫监控的逃脱阶段。这一描述强化了免疫编辑的动态特性与阶段性发展。肠道菌群能够在局部和远端部位影响宿主免疫，调控T细胞等免疫细胞的扩增和分化，发挥对肺癌的免疫调节作用。Jenkins等^[8]^发现肠道菌群失调能降低肺癌肿瘤微环境中肿瘤坏死因子α（tumor necrosis factor α, TNFα）和干扰素γ（interferon γ, IFNγ）的水平，抑制肿瘤细胞凋亡和促进细胞增殖分裂，在细胞间黏附分子-1（intercellular cell adhesion molecule-1, ICAM-1）表达阳性小鼠中促进了肿瘤生长，而补充TNFα可使肿瘤血管中ICAM-1表达升高，增加肿瘤中效应T细胞的数量从而抑制肿瘤生长；长期抗生素治疗能通过CXCL9-CXCR3轴显著降低小鼠肺部自然杀伤（natural killer, NK）细胞和CD8^+^ T细胞数量以及IFNγ、颗粒酶和穿孔素的分泌，影响肺癌的预后。肠道微生物群落不仅具备调控肿瘤免疫监测与抑制抗肿瘤免疫反应的能力，还能介导髓系衍生抑制细胞的募集过程，进而加速癌症的进展。研究^[9,10]^显示核梭杆菌和厌氧胃链球菌能够招募肿瘤浸润性骨髓细胞，增加肿瘤相关巨噬细胞和中性粒细胞，促进肺癌进展。这些研究提示特定的肠道菌群可以通过扩增调节性T细胞和髓系抑制细胞，削弱抗肿瘤免疫反应，进而促进肺癌的发展。

同时，有益菌群通过增强免疫监视功能，如促进NK细胞和T细胞的活性，抑制肿瘤的生长，展现出抗癌潜力。Shoji等^[11]^报道对免疫检查点抑制剂（immune checkpoint inhibitors, ICIs）癌症免疫治疗有反应的非小细胞肺癌患者肠道菌群中经黏液真杆菌含量丰富。目前认为，主要通过两种机制发挥抗癌作用：（1）改善短链脂肪酸（short chain fatty acids, SCFAs）水平，从而塑造肺部免疫力。SCFAs可通过T细胞受体信号激活肺内产生白细胞介素22（interleukin-22, IL-22）的3型天然淋巴细胞（group 3 innate lymphoid cell, ILC3）、调节性T细胞和辅助性T细胞2（T helper 2 cell, Th2），还能够通过激活G蛋白偶联的细胞膜受体，并抑制组蛋白去乙酰化酶的活性实现对免疫系统的精细调节，从而降低肺癌的发病率；（2）肠道菌群可使2型固有淋巴细胞、ILC3、辅助性T细胞17等肠道免疫细胞直接从肠道经血流迁移到呼吸道，影响呼吸系统的免疫活性。除了以上两种机制外，肠道菌群主要还通过CD8^+^ T细胞和CD4^+ ^T细胞介导发挥抗癌作用：总体来说，CD8^+^ T细胞能分泌大量的IFNγ和颗粒酶B，协同作用杀灭致癌细胞。而CD4^+ ^T细胞除了能够补充CD8^+ ^T细胞和NK细胞的细胞溶解活性外，还能通过IFNγ机制直接杀死肿瘤细胞。具体的，双歧杆菌通过调控树突状细胞功能及促进肿瘤微环境内CD8^+^ T细胞积聚的潜力，增强NK细胞分泌IFNγ和穿孔素的能力，展现出显著抗癌作用^[12,13]^。梭状芽孢杆菌通过激活蛋白激酶RNA样内质网激酶，增强CD8^+^ T细胞介导的抗肿瘤免疫，提升三阴型乳腺癌患者的抗肿瘤能力^[14]^。Li等^[15]^进一步研究表明，肠道菌群能够干预树突状细胞内cGAS-STING-IFN-I信号转导途径，进而调控CD8^+^ T细胞的适应性免疫反应，这一过程对肝癌放疗的疗效产生了显著影响。Mager等^[16]^对MB49膀胱癌小鼠模型联合应用肠道微生物代谢物肌苷与CpG后，促进了IFNγ表达阳性的CD4^+^ T细胞在肿瘤微环境中的积聚，增强了小鼠体内细胞毒T淋巴细胞相关抗原4介导的肿瘤杀伤效能，进一步优化了肿瘤预后状况。

### 2.2 炎症调节

慢性炎症在癌症发生发展中起重要作用。研究^[17]^表明，慢性炎症可导致DNA损伤或突变，从而诱导肿瘤细胞的形成并增殖，促进肺癌发生，这就是炎症导致肺癌的“三部曲”。此外，肠道菌群失调可以诱导肿瘤微环境中的促炎因子产生，导致肺癌细胞肿瘤生长、侵袭和转移。Najjary等^[18]^发现与中性粒细胞淋巴细胞计数比值、血小板与淋巴细胞比值和系统免疫炎症指数存在相关性的95个微生物分类群，其中包括与肺癌相关的乳酸杆菌属、链球菌科、真杆菌属和真菌属等。这些菌种通过增加白细胞计数和炎症反应相促进癌症发展。研究^[19]^指出结核杆菌感染会促进肺癌形成，可能机制是持续的结核感染产生TNF，引起肺部炎症和纤维化；最后，肠道菌群也会增加活性氧、甲烷和硫衍生物的反应，也可能有助于肺癌的发生发展。相反，某些有益菌种则可以通过减少促炎因子的产生，抑制炎症反应，减缓肿瘤的发展。Yang等^[20]^研究表明，在非吸烟者群体中增加益生元与益生菌食品的摄入能显著降低肺癌发病风险。作用机制是益生元与益生菌促进肠道IL-10的产生，同时减少促炎因子IL-1β及IL-6的释放。Chen等^[21]^发现嗜黏蛋白阿曼克菌（*Akkermansia muciniphila*, Akk）联合顺铂可通过下调Ki67、p53、FasL蛋白水平和上调Fas蛋白水平，诱导IFNγ、IL-6、TNFα等抗癌因子产生，抑制CD4^+^ T细胞、调节性T细胞、Foxp3调节性T细胞的表达，从而降低肿瘤体积增加的速度，改善肿瘤病理形态的变化；而Akk与增生平的联合使用则能增加血清IL-1β、IL-6、TNFα水平，增加结肠和支气管肺泡灌洗液中分泌性免疫球蛋白A浓度，最终提高脾脏和胸腺指数以及显著降低肺癌肿瘤重量^[22]^。由肠道菌群产生的大蒜二烯丙基三硫化物显著降低亚硝胺物丁酮诱导的p65和p-p65蛋白的表达并且增加IκBα蛋白的表达，减少促炎因子的产生，从而阻止肿瘤炎症微环境的形成^[23]^。

## 3 肠道菌群对代谢和治疗效果的影响

### 3.1 细胞代谢的影响

肿瘤细胞的代谢特性与正常细胞显著不同，肠道菌群通过影响宿主的代谢通路可能在肿瘤的发生和进展中发挥作用。与正常人群相比，肺癌患者细胞的代谢通路发生了显著变化，这提示肠道菌群在调整代谢抑制肿瘤生长方面可能具有潜力。Zheng等^[24]^研究发现与健康人群对照组相比，肺癌患者肠道菌群在细胞抗原处理、类固醇生物合成、泛素系统介导的蛋白降解、转录因子相关蛋白活性、胆汁酸分泌机制及线粒体脂肪酸链延长等多个代谢领域呈现出增强的趋势；相反，与细菌运动蛋白的效能、细菌趋化行为、黄酮及黄酮醇类化合物的合成路径、细胞凋亡的调控以及G蛋白偶联受体介导的信号通路相关的代谢活动则显著减少。肠道菌群主要通过以下两种方式导致肿瘤代谢表观遗传学的改变：（1）直接改变用于表观遗传修饰的底物池；（2）产生其他化合物，间接改变参与表观遗传修饰酶的活性。具体来说，灵芝多糖、绞股蓝皂苷和薄叶草提取物通过增强细胞代谢，促进细胞SCFAs（如丁酸盐和丙酸钠）产生使肿瘤的细胞周期阻滞，特别是G_2_/M期阻滞和细胞凋亡，诱导肿瘤细胞凋亡^[25⇓⇓⇓-29]^。人参多糖增强肠道微生物介导的戊酸生成路径，减少犬尿氨酸与色氨酸之间的比例失衡，增强其抗癌效应^[30]^；附子通过调节肠道菌群的戊糖磷酸途径来减低肿瘤细胞的三羧酸循环，抑制了肿瘤生长^[31]^。这些研究提示肠道菌群的代谢活动可能成为肺癌治疗的靶点。

近年来发现肠道菌群能够通过代谢产物改变肺癌肿瘤代谢重编程进程，由于肿瘤细胞内有氧糖酵解增加的乳酸产生，胞内pH值降低，使之处于酸中毒环境，促进组蛋白脱乙酰化。此外，乙酰辅酶A（acetoacetyl coenzyme A, Ac-CoA）又可以刺激启动子组蛋白乙酰化。在葡萄糖缺乏的条件下，Ac-CoA相对于辅酶A的比值下降，影响组蛋白乙酰化水平，癌基因又可能通过Ac-CoA代谢改变染色质的生长，从而影响肺癌肿瘤细胞的分裂、分化与增殖。另外，神经胶质瘤和前列腺肿瘤中丝氨酸/苏氨酸激酶磷酸化水平与组蛋白乙酰化的整体水平显著相关；癌基因*MYC*也被证明是Ac-CoA代谢和组蛋白乙酰化的关键调节剂。因此，上述两个方面在生成Ac-CoA和促进组蛋白乙酰化方面具有重要作用。

### 3.2 肿瘤治疗效果的影响

肠道菌群可通过影响免疫治疗药物的代谢路径，进一步增强其抗癌效果，并且肠道菌群可以通过调节宿主的免疫反应增强ICIs的疗效。研究^[32]^表明乳酸菌、梭状芽孢杆菌和菌间球菌是与非小细胞肺癌患者ICIs疗效呈正相关的肠道微生物群，而嗜胆菌、萨特菌和副拟杆菌则呈负相关。有研究^[33]^发现活性肠球菌分泌产生免疫活性多肽的NIpc/p60肽聚糖水解酶分泌抗原A的同源物，在小鼠模型中改善了ICIs的抗肺癌效果。肠道菌群增强ICIs疗效的主要机制包括:（1）微生物病原相关分子模式（pathogen-associated molecular patterns, PAMPs）与树突状细胞反应，可能导致先天免疫激活、释放包括IL-12在内的多种细胞因子和趋化因子；（2）细菌抗原与某些肿瘤抗原的分子模拟可能导致免疫激活；（3）肠道细菌产生的小分子调控子刺激CD8^+^ T细胞。而在抗程序性死亡受体1（programmed cell death protein 1, PD-1）治疗中，变形菌门、厚壁菌门、拟杆菌门、鼠李糖乳杆菌亚种和放线菌等菌落可以提高抗PD-1治疗的疗效^[34⇓-36]^，原因是肠道放线菌等的次生代谢产物显著增加了肺癌患者体内外周记忆T细胞与NK细胞的数量，并增加CCR9^+^ T细胞、CXCR3^+^ T细胞和CD4^+^ T细胞的募集，以IL-12依赖的方式提高PD-1受体阻断的疗效^[37⇓-39]^；另外，肠球菌原噬菌体可以诱导接受抗PD-1药物治疗后的小鼠肿瘤中卷尺蛋白（tape measure protein, TMP）交叉反应抗原的表达，通过TMP表位特异性的H-2K（b）限制CD8^+^ T细胞反应发挥抗癌作用^[40]^。

在化疗药物、靶向药物甚至中成药中肠道菌群也能显著增加其疗效，双歧杆菌和化疗药物奥沙利铂协同作用降低了Treg细胞群，同时增加了脾脏和肺癌肿瘤中的CD4^+^ T细胞、CD8^+^ T细胞、效应CD8^+^ T细胞和NK细胞，使小鼠中IFNγ和IL-2显著增加、TNFα和IL-10显著降低，抑制肿瘤生长^[41]^。在小鼠肺癌模型中卵形拟杆菌和溶木拟杆菌可诱导CXCL9和IFNγ表达，显著提高靶向药物厄洛替尼的疗效^[42]^。西黄丸是一种经典的抗癌中药配方，能增加有益菌拟杆菌属和*g-norank-f-Muribaculaceae*属的比例，联合安罗替尼后增加了Lewis肺癌小鼠抗肺癌作用，其作用是抑制血管相关信号通路，导致血管生成蛋白表达下调，最终使肿瘤血管的管径和大小减少，有效抑制了肿瘤的生长^[42]^。

## 4 展望

肠道菌群的失调不仅影响肺癌的发生和发展，在治疗中的重要性也已经得到初步证实。未来研究应着力于深入探究肠道菌群对肺癌发生机制的影响，尤其是在免疫调控和代谢调节方面。同时，肠道菌群作为治疗靶点的临床应用前景广阔，有望为肺癌个性化治疗提供新思路。为此，研究者可对患者的肠道菌群进行全面检测与分析，明确其组成与特点；然后根据菌群情况选择合适的干预手段，比如通过粪菌移植、饮食调整或补充益生元等方式，有针对性地调节患者肠道菌群。在此过程中还需密切监测治疗效果，并根据实际情况灵活调整治疗方案。而如何精准地检测和分析患者的肠道菌群，并明确其与肺癌的具体关联，以及如何确保治疗方案的安全性和长期稳定性等方面仍需进一步研究和探索。
